# Drug Repositioning and Subgroup Discovery for Precision Medicine Implementation in Triple Negative Breast Cancer

**DOI:** 10.3390/cancers13246278

**Published:** 2021-12-14

**Authors:** Zainab Al-Taie, Mark Hannink, Jonathan Mitchem, Christos Papageorgiou, Chi-Ren Shyu

**Affiliations:** 1 Institute for Data Science & Informatics, University of Missouri, Columbia, MO 65211, USA; zsa36f@mail.missouri.edu (Z.A.-T.); mitchemj@health.missouri.edu (J.M.); 2 Department of Computer Science, College of Science for Women, University of Baghdad, Baghdad 10070, Iraq; 3 Department of Biochemistry, University of Missouri, Columbia, Missouri, MO 65211, USA; HanninkM@missouri.edu; 4 Department of Animal Sciences, Bond Life Sciences Center, University of Missouri, 1201 Rollins Street, Columbia, MO 65211, USA; 5 Department of Surgery, School of Medicine, University of Missouri, Columbia, MO 65212, USA; 6 Department of Research Service, Harry S. Truman Memorial Veterans’ Hospital, Columbia, MO 65201, USA; 7 Electrical Engineering and Computer Science Department, University of Missouri, Columbia, MO 65211, USA; 8 Department of Medicine, School of Medicine, University of Missouri, Columbia, MO 65212, USA

**Keywords:** subgrouping, patient stratification, triple negative breast cancer, ferroptosis, antioxidant, drug repositioning, drug repurposing, data mining, network analysis, explainable artificial intelligence

## Abstract

**Simple Summary:**

The heterogeneity of complicated diseases like cancer negatively affects patients’ responses to treatment. Finding homogeneous subgroups of patients within the cancer population and finding the appropriate treatment for each subgroup will improve patients’ survival. In this study, we focus on triple-negative breast cancer (TNBC), where approximately 80% of patients do not entirely respond to chemotherapy. Our aim is to find subgroups of TNBC patients and identify drugs that have the potential to tailor treatments for each group through drug repositioning. After applying our method to TNBC, we found that different targeted mechanisms were suggested for different groups of patients. Our findings could help the research community to gain a better understanding of different subgroups within the TNBC population and can help the drugs to be repurposed with explainable results regarding the targeted mechanism.

**Abstract:**

Breast cancer (BC) is the leading cause of death among female patients with cancer. Patients with triple-negative breast cancer (TNBC) have the lowest survival rate. TNBC has substantial heterogeneity within the BC population. This study utilized our novel patient stratification and drug repositioning method to find subgroups of BC patients that share common genetic profiles and that may respond similarly to the recommended drugs. After further examination of the discovered patient subgroups, we identified five homogeneous druggable TNBC subgroups. A drug repositioning algorithm was then applied to find the drugs with a high potential for each subgroup. Most of the top drugs for these subgroups were chemotherapy used for various types of cancer, including BC. After analyzing the biological mechanisms targeted by these drugs, ferroptosis was the common cell death mechanism induced by the top drugs in the subgroups with neoplasm subdivision and race as clinical variables. In contrast, the antioxidative effect on cancer cells was the common targeted mechanism in the subgroup of patients with an age less than 50. Literature reviews were used to validate our findings, which could provide invaluable insights to streamline the drug repositioning process and could be further studied in a wet lab setting and in clinical trials.

## 1. Introduction

Breast cancer (BC) is the leading cause of death among female patients with cancer [1,2]. BC is a highly heterogeneous disease. BC has intertumoral heterogeneity, where the tumor is heterogeneous among the BC patients, and intratumor heterogeneity, where the tumor is heterogeneous within the tumor of an individual patient [3]. A consequence of heterogeneity is that patients with breast cancer can have different reactions to the same drug. BC is a clear example of the need to have personalized medicine that is patient-centric rather than disease-centric. Implementing precision medicine in a healthcare system requires developing patient stratification methods to find homogeneous subgroups of patients to tailor drugs to these subgroups. Developing de novo drugs is time-consuming, taking between 10 and 15 years, and is expensive, costing about $1.6 billion [4,5]. To overcome the problems associated with developing drugs for a subpopulation of a disease, drug repositioning (DR), the redirection of already approved drugs to be used for additional diseases by finding new indications, emerges as an alternative to support precision medicine implementation [6].

Different molecular features have been used to stratify patients and reposition drugs. These molecular features range from using a single gene to using heterogeneous molecular data types; for example, using P53 to stratify patients based on their P53 status and using the RACK1 status to determine personalized treatment [7,8]. Moreover, heterogeneous molecular data was used for cancer subtyping and the repositioning of drugs after ranking genes based on the Gene Ontology (GO) pathways analysis [9]. The transcriptional response to drugs was used to infer signaling interactions and to reposition drugs [10]. Drug repositioning was also accomplished by targeting pathways that play a role in cancer proliferation and progression [11]. The oncogenic PI3K-dependent inhibitor was recommended as a cancer therapy [12]. Drugs were recommended for repositioning based on the similarity between a particular cancer type therapy and a given drug using a drug-pathway network [13]. The association between gene mutation and expression was also used to guide the drug discovery process [14]. A network analysis was used widely to reposition drugs for different types of cancer. Studies have introduced many heterogeneous network models to reposition drugs, including the drugs–genes–diseases network [15,16], and drug–targets–pathways–genes–diseases, where the association between drugs and diseases was found based on multiple targeted genes and pathways between drugs and diseases [17]. The drug-disease association was also inferred based on a set of heterogeneous networks including drug–gene, disease–gene, protein–protein, and gene co-expression networks to find drug-disease associations and to reposition drugs [18]. 

Several methods have been developed to understand the underlying mechanism of BC and the recommend drugs. A gene expression analysis was used to identify pathways involved in BC invasion and potential drug targets [19]. In the context of DR, drugs have been recommended for their antiproliferation effect on BC patients [20]. Additionally, drugs that share side effects with BC therapies have been recommended to be repositioned for BC [21]. A pathway analysis was used with single-cell data to recommend drugs for BC [22]. A network analysis also was used to reposition drugs for breast cancer. A tissue-specific protein–protein interaction (PPI) network was used to evaluate and reposition drugs over the drug–miRNA–diseases network [23]. A network propagation analysis was used to reposition drugs based on a drug-pathway network, where pathways for each drug were identified by an enrichment analysis of the genes regulated by each drug using the Connectivity Map (CMAP) for the drug’s phenotypic profile [24]. Moreover, computational methods with diverse molecular data types were developed to identify therapeutic targets for BC [25]. These methods considered breast cancer in general without considering its subtypes and did not address the heterogeneity of BC. Other methods have been developed to stratify BC patients. This stratification was done based on cell receptor statuses; for example, an estrogen receptor positive (ER+) status and an estrogen receptor negative (ER−) status [26].

Based on the gene expression profile, four molecular subtypes have been identified for breast cancer which are luminal A, luminal B, human epidermal growth factor receptor 2 (HER2)-enriched, and basal-like (triple negative) [27]. Computational methods have been developed to find therapeutic biomarkers and to reposition drugs for breast cancer subtypes. The correlation between DNA copy number alterations and gene expression was used to find biomarkers for each subtype [28]. Electronic health record (EHR) and differentially expressed gene (DEG) data were used to reposition drugs for the four BC molecular subtypes by finding drug pairs using drug–protein relations [29]. mRNA was used to reposition drugs for these subtypes using gene co-expression [30]. Moreover, drugs were predicted based on the suggested miRNA biomarkers for each subtype [31]. Drugs were recommended based on the number of hub genes each drug targeted within the miRNA–protein–drug network [32]. An integrated network of relations between lncRNA, miRNA, and mRNA was used to recommend drugs that reversed the lncRNA expression of each BC molecular subtype [33]. A pathway analysis was also used to reposition drugs for BC subtypes [34]. Biomarker predictions using cell line data were used to stratify BC patients and suggested drugs for each subgroup [35]. These methods have demonstrated the importance of stratifying BC patients into homogeneous subgroups and repositioning drugs into these subgroups. Still, they have considered only the genotypic characteristics of BC patients without considering the significance of phenotypic features. Other methods have taken phenotypic data into consideration, but the majority of these methods include the phenotypic features as the post-analysis of the stratification process, where clustering methods have been used to cluster the genotypic profiles of patients and then map the phenotypic traits onto each cluster in order to assign patients to each subgroup [36].

This study implements our data-driven approach to stratify BC patients based on genotypic and phenotypic data. The genotypic data is mapped onto a heterogeneous drug knowledge base to find druggable homogeneous BC subgroups and to reposition drugs for each subgroup [37]. We have decided to focus on triple negative breast cancer (TNBC), as defined by the lack of Estrogen Receptors (ERs) and progesterone receptors (PRs) and by a HER2-negative status, due to its increasingly recognized heterogeneity not only on the molecular level but also on the pathologic and clinical levels [38]. The lack of targets like ER, PR, and HER2 in TNBC implies that chemotherapy remains the only treatment of choice for patients with TNBC, which unfortunately fails to achieve prolonged remission in the majority of cases. Indeed, on recurrence, patients with TNBC have worse survival outcomes than patients with ER-positive and/or HER2-positive BC subtypes [39,40]. Other studies have demonstrated the importance of finding homogeneous subpopulations within the TNBC population, but these studies mainly focused on a set of genes or single nucleotide polymorphisms (SNPs) to identify drug targets for TNBC [41,42]. Other methods had a broader range of data, such as gene expression data, to identify key genes that could become drug targets [43]. As these methods represent a step toward understanding TNBC progression and improving patient survival, targeting a heterogeneous disease requires considering a wide variety of genotypic and phenotypic factors. Therefore, we propose to use a data-driven approach in order to dissect TNBC heterogeneity as much as possible and to discover druggable molecular targets that may enhance the chemotherapy benefit by blocking chemoresistance pathways and, thus, cancer recurrence in TNBC patients.

## 2. Materials and Methods

**Figure 1:**
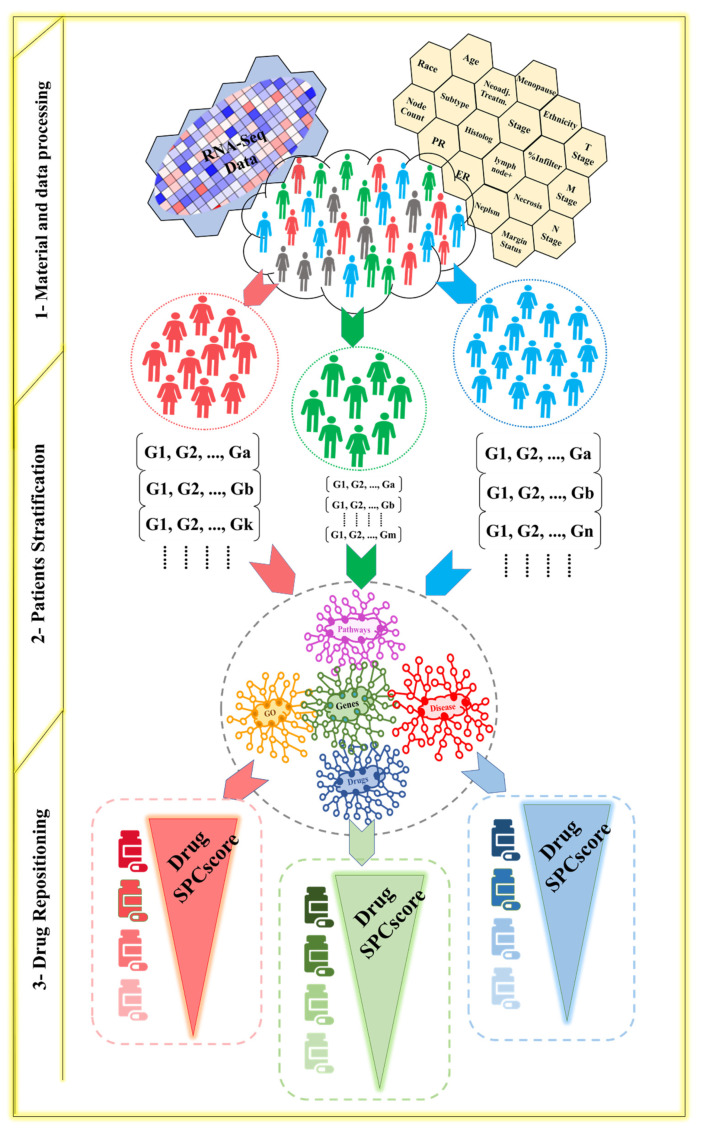
Flowchart of the data-driven drug repositioning process using phenotypic and genotypic breast cancer data of TCGA, patient stratification methods, and a knowledge base for recommendation of drug repositioning.

Data from 980 breast cancer patients was obtained from The Cancer Genome Atlas (TCGA). The dataset consisted of phenotypic data and genotypic data. For the phenotypic variables, we used 19 clinical variables (Figure 1, Module 1 and Supplementary Figure S1: Table S1). The continuous phenotypic variables were converted into categorical variables. The categorization of the clinical variables was done based on medical guidelines [44]. The genotypic variables were the RNA-seq data of these patients. 

A differential analysis was done between 94 normal and 980 tumor samples. The normal and tumor data were downloaded from TCGA. Based on the differential analysis done using EdgeR, we selected 1531 of the most differentially expressed genes with a *p*-value less than 0.05, a log2FC > +2 for upregulated genes, and a log2FC < −2 for the downregulated genes. Then, the genotypic data were normalized using log2 and categorized based on the z-score value for each gene in each patient. These genes were categorized into upregulated (z-score > 1), downregulated (z-score < 1), and normal (z-score between 1 and −1) genes. The drug repositioning knowledge base (DR-KB) was represented as a neo4j graph with a gene-centric schema, including relations between genes, pathways, molecular function, cellular components, biological processes, disease, and drugs (Figure 1, Module 2). The relations between these biomedical entities and the source databases of these relations can be found in Supplementary Figure S1: Table S2 [45]. 

The method consisted of two parts. The first part was the identification of the homogeneous subgroups within a heterogeneous disease population. To find the homogeneity between patients in a subgroup, genotypic pattern mining was performed for patients sharing phenotypic features. The relations between gene patterns and biomedical entities were extracted from the DR-KB after mapping the gene patterns to the DR-KB graph. The second part of the method was to find suitable drugs for each subgroup by developing and applying a drug repositioning algorithm. This algorithm used a graph analysis to analyze each subgroup’s network. The drugs in each network were then ranked according to various factors, as discussed below. 

### 2.1. Patient Stratification

To enable precision medicine, we stratified BC patients into homogeneous subgroups. Each subgroup is defined by a set of clinical variables and genotypic patterns common among the patients and representing the underlying factors that are unique to a subgroup and that differentiate it from the rest of the population. The patients in a given subgroup should have a statistically significant difference from the outer population based on gene expression patterns, defined as the co-occurrence of genes which have certain regulation levels. For example, if two genes are upregulated or downregulated in most patients within a subgroup, these genes are considered a pattern in that subgroup. In our data mining and network analysis method, gene–gene interactions and enrichment analysis are used to contrast a subgroup and the outer population instead of using only gene patterns. The genotypic information is then integrated into a network. Neo4j is used to do the network analysis on top of the contrast pattern mining that is performed on the genotypic data. 

We represent our subgroup stratification method as a three-level framework. These levels are path expansion, floating subgroup selection, and inclusion and exclusion. The subgrouping algorithm is an extension of our exploratory data mining methods [37,46]. The algorithm begins with a disease population. The patient stratification process then divides the population into subgroups based on categorical values of the clinical variables. To consider a subgroup as valid, the subgroup should significantly differ from the outer population. The algorithm adds clinical variables to the current subgroup to create a more focused subgroup. The process starts with one categorical value for a clinical variable which is expanded by adding more variables, as long as there is a statistical significance of doing so and a better contrast than the previous step. This process is similar to searching among different paths to reach the local optimum solution. In our method, the local optimum solution is a subgroup with the highest contrast from the outer population. For example, if the algorithm finds that patients with the TNBC subtype have the highest contrast from the outer population, it will add another variable, such as invasive ductal carcinoma (IDC), as a histological type. This creates a more focused subgroup consisting of two variables, TNBC and IDC. 

The algorithm checks different variables in parallel. We refer to this process as checking different paths, where each path represents a different set of variables that define a subgroup. The path expansion processes expand the search for subgroups with a high contrast to the outer population by considering the top X paths with high contrast scores. 

To consider a subgroup valid so that the algorithm continues adding a variable in that path, a contrast score is calculated. That score is based on gene expression patterns in each subgroup and the interactions between genes; the pathways, molecular functions, cellular components, and biological processes to which each gene belongs; diseases; and drugs that affect the gene expression patterns (Figure 1, Module 2 and Equation (4)). The patterns of a subgroup should be frequent in that subgroup. Support [47] (Equation (1)) is used to determine if a pattern is frequent based on a user-defined threshold. In addition to a pattern being frequent in a subgroup, it must have a high contrast in the given subgroup compared to the outer population. The growth rate [48] (Equation (2)) is used to evaluate the contrast of a pattern. The support and growth rates are used as parameters to calculate the gene expression pattern contrast score of a subgroup. We have developed a *J*-value [46] (Equation (3)) to calculate the contrast for all patterns in a given subgroup.

(1) $$Support\left(p,D\right)=\frac{\left|\langle D,p\rangle \right|}{\left|D\right|},$$

where *p* is a pattern, *D* is the patients’ data in the dataset, |<*D*,*p*>| is the number of patients who have this pattern, and |*D*| is the total number of patients in the dataset.

(2) $$Growth\;\left(cp,\;S_{G1},\;S_{G2}\right)=\frac{Max\left\{s_{1},s_{2}\right\}}{Min\left\{s_{1},s_{2}\right\}},$$

where *cp* is the contrast pattern, *S_G_*_1_ is the focus subgroup, *S_G_*_2_ is the outer population, *s*_1_ is the support of *cp* in the focus subgroup, *s*_2_ is the support of *cp* in the outer population.

(3) $$J\text{-}\mathrm{value}=\frac{T\;*\;J_{org}+M\;*\;{\bar{J}}_{avg}}{T+M},\;$$

where T is a parameter related to the population size preference that depends on the type of the disease under study and, whether it is a rare disease or not, based on the concept of the Bayesian average [49,50,51]. 

Before doing path expansion, the algorithm randomly picks a set of subgroups and calculates the average population size, M, of these subgroups. $J_{org}$ and $J_{avg}$ are calculated based on the initial *J*-value, which is $J^{2}\leq \underset{i\leq J}{\sum }Growth\left(cp,\;S_{G1},S_{G2}\right)$. J-value is a quantitative index used to evaluate the overall quality of a set of contrasting patterns in the subgroup. In addition to J-value, a network analysis is used to calculate the network contrast score (*NCS*) for each candidate subgroup (Equation (4)).

(4) $$NCS=1-\frac{\sum_{i=1}^{n}\frac{E_{i,1}\cap E_{i,2}}{E_{i,1}\cup E_{i,2}}}{n},\;$$

where *E_i_*_,1_ is biomedical entity, type *i*, in the focus group’s network; and *E_i_*_,2_ is biomedical entity, type *i,* in the outer population network. These biomedical entities are the genes and the other biomedical entities that affect these genes like the disease, the drug, and the biomedical entities to which these genes are part of, like pathways, molecular function, cellular components, and biological processes.

*NCS* is the score of contrast between the network of the subgroup of interest and the outer population network. Mapping the gene patterns to the DR-KB creates a heterogeneous network of different biomedical entities. In this work, these biomedical entities include genes, pathways, cellular functions, molecular components, biological processes, diseases, and drugs. The subgroup contrast score (*SPCscore*) [37] (Equation (5)) is calculated for each subgroup.

(5) $$SPC_{Score}=J\text{-}\mathrm{value}\;*\;NCS,$$

The output subgroups are filtered based on a user-defined threshold for the $SPC_{Score}$. A set of subgroups with high contrast is selected for further study. This study focuses on five TNBC subgroups that have a statistically significant contrast from the outer population. These subgroups are the input for the next step in which a computational algorithm is used to reposition drugs for each subgroup.

### 2.2. Drug Repositioning

A drug repositioning algorithm is used to identify candidate drugs for each subgroup. As mentioned previously, a subgroup network consists of heterogeneous entities represented as a neo4j graph, G = (V_G_, E_G_). In each network, there are a number of entities of each type, including drugs. To find the drugs with the highest potential to treat the patients in a subgroup, the drugs within each network are ranked according to their relevance to the genotypic characteristics (Figure 1, Module 3). We have developed a drug scoring function that includes different factors to put the drug impacts within the subgroup network (SGNW) into perspective, as well as their impacts in general on human cells. We consider the number of abnormally expressed genes in a subgroup, gene expression affected by each drug, and the importance of each gene within the SGNW. For example, hub genes have more significance than genes with few connections. Gene weight is based on the percentage of entities of each biomedical type that connects to each gene. The mean of gene frequency (*MGF*) is calculated for each gene in the SGNW, as follows:

(6) $$MGF\left(Gene_{i}\right)=\frac{\sum_{k=1}^{e}\frac{nE_{ki}}{NE_{k}}}{e},$$

where $Gene_{i\;}$ is a gene in the SGNW for which frequency is calculated, e is the total number of entity types in S, and $E_{k}\in \;S$, S=[Gene, Pathway, Biological process, Cellular component, Molecular function], and $nE_{ki}$ are the total number of entities of type $E_{k}$ that are in direct interaction with $Gene_{i}$. $NE_{k}$ is the total number of entities of type $E_{k}$ that exist in the SGNW. The MGF is calculated for all the genes in the SGNW.

For each drug, the algorithm calculates an initial weight, which is the accumulative gene frequency (*AGF*), corresponding to the accumulative weight of all the genes connected to each drug in the SGNW.

(7) $$AGF\left(Drug_{j}\right)=\sum_{i=1}^{m}MGF\left(Gene_{i}\right),$$

where *m* is the total number of genes whose expression is altered by $\;Drug_{j}$ within a given subgroup. The AGF for $Drug_{j}$ is the overall summation of the MGF scores for each of these genes. This equation assigns a value that represents the importance of the drugs in the context of their ability to perturb gene expression. The weight represents the importance of a gene in the subgroup network. The AGF value characterizes a drug’s importance as the average of the genes’ importance connected to that drug. 

The other significant factor to consider is the patterns of each subgroup. In our algorithm, we are not looking at the genes as independent entities. In addition to taking the interaction of the subgroup genes with other genes and other biomedical entities, we consider the gene patterns formed within each subgroup. To address this, we calculate the percentage of patterns targeted by each drug, $PA\left(Drug_{j}\right)$, as follows:

(8) $$PA\left(Drug_{j}\right)=\frac{NP_{j}}{NP},$$

where $NP_{j}$ is the total number of gene expression patterns targeted by $Drug_{j}$ in the subgroup of interest, and NP is the total number of gene expression patterns in this subgroup. Then, the overlap score measure (*OSM*) is calculated for each drug in the SGNW. The OSM of a drug is the summation of that drug’s importance on two levels. One level is the connection between that drug and the genes in the subgroups, AGF. The other level is the importance of the drug based on the patterns targeted by that drug, PA:

(9) $$OSM\left(Drug_{j}\right)=PA\left(Drug_{j}\right)+AGF\left(Drug_{j}\right).$$

In addition to the accumulative gene weights and the percentages of gene patterns impacted by each drug, the algorithm considers the ratio of genes whose expression is affected by each drug. This is accomplished by calculating gene percentages for each drug (*GP*) as follows:

(10) $$GP\left(Drug_{j}\right)=\frac{NG_{j}}{NG},\;$$

where $NG_{j}$ is the total number of genes whose expression is perturbed by $Drug_{j}$ in the subgroup of interest, and NG is the total number of genes in this subgroup.

We also must ensure that the drugs with high a OSM are essential to a given subgroup. It is similar to the idea in the information retrieval theory, where terms that appear in the majority of documents have less importance, like stop words such as “the”, and “is”, and so on. We were inspired by the idea of the inverse document frequency (*IDF*) to calculate the drug score [52]. The inverse drug frequency (*IDF*) is calculated for each drug in each subgroup. This helps to increase the score of drugs that are unique to a subgroup and decrease the score of common drugs that could be related to cancer in general, but not to that subgroup of interest in particular. The biological reasoning behind using *IDF* as a scoring factor is to account for the amount of perturbation a drug could cause in the human cells. We want to select those drugs that specifically perturb several subgroups’ gene patterns. The *IDF* is calculated as:

(11) $$IDF\left(Drug_{j}\right)=\log_{10}\left(\frac{Ns}{nS_{j}}\right),\;$$

where Ns is the total number of genes in the RD-KB and $nS_{j}$ is the number of genes targeted by $Drug_{j}$ in that subgroup network.

Finally, the impact of each drug is evaluated within the SGNW to rank the drugs based on different factors that determine each drug’s effect. The final drug score ($D_{Score}\left(Drug_{j}\right)$) is:

(12) $$D_{Score}\left(Drug_{j}\right)=OSM\left(Drug_{j}\right)\;*\;IDF\left(Drug_{j}\right)*\;GP\left(Drug_{j}\right).\;$$

Each drug within the SGNW is assigned a $D_{Score}$ for ranking purposes. The higher the score, the more potential the drug has as a treatment for a given subgroup. Our method addresses the importance of considering different factors in ranking drugs. The ranking is not only based on the genes connected to the drugs and their significance in the network, but also on the gene expression perturbation a drug can cause in human cells and the number of gene patterns affected by the drug within the subgroup’s network.

## 3. Results

Patients with triple-negative breast cancer (TNBC), who have negative immunohistochemical staining for estrogen receptors (ER), progesterone receptors (PR), and human epidermal growth factor receptor 2 (HER2) [53], have the lowest survival rate and highest mortality within BC patients [54,55]. Patients with TNBC have a heterogeneous response to therapy, in which approximately 80% of patients do not completely respond to chemotherapy [56]. The importance of finding better treatments for TNBC has been demonstrated in many studies. Multi-omics data and a network analysis was used to repurpose drugs for TNBC [13]. The network analysis was used to find modules active in TNBC, but not in normal BC or other BC subtypes. Transcriptional and interactome data were used to create a PPI network to identify highly connected modules and hub genes as candidate drug targets for repurposed therapies [13]. Multi-target drugs were also considered for repositioning to TNBC after creating a drug–target network with the integration of pathway information and the identification of protein–protein interactions [57]. Our study offers new guidance in considering heterogeneity within subgroups and the importance of finding subpopulations within TNBC. Moreover, in addition to using the direct relationship between drugs and genes in TNBC, our approach leverages other factors, like the perturbation that the drug can cause in the cell. 

### 3.1. Subgroups and Drugs Analysis

Different criteria were used to filter and select subgroups. The support of the subgroup patterns was greater than 70%, the growth was greater than 1.5, and the confidence was 1. The resulting subgroups were ranked based on their SPCscore. The subgroups with an SPCscore > 0 were retained for further filtering and analysis because an SPCscore = 0 means there is no significant contrast between a given subgroup and the outer population. After filtering the subgroups based on their contrast score and the biomedical importance to our research question, the resulting subgroups were five TNBC subgroups with the patients’ neoplasm subdivision, race, and age as clinical variables. In this work, after analyzing the top drugs for these subgroups, we found ferroptosis as a common cell death mechanism. Ferroptosis is regulated cell death (RCD). It is driven by lipid peroxidation and has an iron dependency [58]. In the subgroups below, we found more than one drug within the top five drugs that plays a role in inducing ferroptosis. This result suggests that focusing on ferroptosis in treating TNBC may be a novel therapeutic route and may be advantageous for these patients. We found the ferroptosis-inducing drugs were suggested for the subgroups with race and neoplasm subdivision as phenotypic variables. Still, there were no ferroptosis-inducing drugs suggested for the younger subgroups with an age below 50. This finding coincides with the previous finding of ferroptosis being negatively correlated with age [59]. The suggested subgroups are as follows:

- *Subgroup1* is for patients who are Black or African-American with no history of a neoadjuvant treatment. This subgroup has 30 patients, and it has 286 unique DEGs to this group (Tables S3 and S4). The drugs that can induce ferroptosis that were suggested for this subgroup are Afatinib, Gefitinib, Bosutinib, Lapatinib, and Fulvestrant.
  - Afatinib was found to be a ferroptosis inducer in TNBC by targeting EGFR [59]. Afatinib’s mechanism of action includes binding to wild-type epidermal growth factor receptors (EGFR) and irreversibly inhibiting the kinase activity of all ErbB family members, which are well-recognized as an oncogenic driver in epithelial cancers. Thus, Afatinib can inhibit the proliferation of cancer cell lines [60]. Analyzing the patient profiles in this subgroup showed that EGFR was abnormally expressed in this subgroup. ErbB4 had an abnormal expression as well.
  - Gefitinib is an epidermal growth factor receptor (EGFR) and a tyrosine kinase inhibitor. It has antiproliferative and anti-tumoral activity in BC [61,62]. Gefitinib targets EGFR and is used to induce ferroptosis [59]. The proliferation of TNBC cells can be inhibited by targeting EGFR kinase activity using gefitinib [63]. Moreover, it can be used as a combined therapy with multiple EGFR-TKIs, such as lapatinib and erlotinib, to overcome the resistance to EGFR-targeted therapy in TNBC. The inhibition of EGFR, in combination with the dual inhibition of cdc7/CDK9, results in reduced cell proliferation, accompanied by the induction of apoptosis, the G2-M cell cycle arrest, the inhibition of DNA replication, and the abrogation of CDK9-mediated transcriptional elongation in TNBC cells [63]. Using gefitinib for this subgroup will inhibit EGFR and cdc7 that was upregulated in this subgroup. Inhibiting them is necessary to reduce cell proliferation. CDK1 and CDK5, which are cyclin-dependent kinases (CDKs), were found to be upregulated in this subgroup. These protein kinases control cancer progression and inhibiting them prevents cancer proliferation [64].
  - Bosutinib promotes autophagy and research has shown that there is crosstalk between autophagy and ferroptosis [65]. BCR-Abl is the target for bosutinib, dasatinib, imatinib, nilotinib, ponatinib, and regorafenib. Bosutinib is a dual Src/Abl inhibitor [66]. In this subgroup, Src was upregulated; therefore, this drug will inhibit Src and reduce cell proliferation. Src belongs to a different set of mechanisms associated with cancer progression, like inducing a metastatic phenotype, enhancing tumor growth, and enhancing angiogenesis [67].
  - Lapatinib is a tyrosine kinase inhibitor. This drug was used as a combined therapy with siramesine, which is a lysosome disrupting agent, to induce ferroptosis and reactive oxygen species (ROS) in TNBC [68]. Lapatinib is an inhibitor of ErbB1 and ErbB2 and induces ferroptosis in BC cell lines by altering iron regulation. It is used as a combined therapy to induce ferroptosis in TNBC by inhibiting the iron transport system, leading to an increase in ROS and cell death [69]. Ferroportin-1 (FPN) is an iron transport protein responsible for the removal of iron from cells. Its expression decreases after treatment with siramesine in combination with lapatinib. The overexpression of FPN decreases ROS and cell death, whereas the knockdown of FPN increases cell death after siramesine and lapatinib treatment. This indicates a novel induction of ferroptosis through altered iron regulation by treating breast cancer cells with a lysosome disruptor and a tyrosine kinase inhibitor [68]. Lapatinib is a tyrosine kinase inhibitor of the epidermal growth factor receptor (EGFR) and ErbB2 (HER2) tyrosine kinases. Studies in vitro showed that lapatinib inhibited the proliferation of ErbB2 and EGFR, which are overexpressed in cancer cells [70,71]. Siramesine and lapatinib gave the best combination index, and this combination induced ferroptosis through iron-mediated ROS and the downregulation of heme oxygenase 1 (HO-1) levels [72]. In TNBC, siramesine and lapatinib increases Transferrin Receptor (TFRC) expression and decreases ferroportin1 (FPN1) expression, thus elevating the level of intracellular iron [73].
- *Subgroup2*: The patients in this subgroup are not Hispanic or Latino and White; they have less than three positive lymph nodes, and their histological type is an intraductal carcinoma. This subgroup has 37 patients and 87 unique DEGs that are abnormal in this subgroup, but not in the other subgroups (Tables S3 and S4). The drugs that can induce ferroptosis that are suggested for this subgroup are fluvastatin, lovastatin, and gefitinib.
  - Fluvastatin targets HMG-CoA and can induce ferroptosis by decreasing the expression of glutathione peroxidase 4 (GPX4). This effect is time- and concentration-dependent [74,75]. Treatment with this statin induces ferroptosis by inhibiting GPX4 and the key products in the mevalonate pathway, like 3-hydroxy-3-methyl-glutaryl-coenzyme A reductase, and the depletion of CoQ10, that reduces the levels of this key membrane antioxidant [74,76,77]. Fluvastatin can be used as a combined therapy with RSL3, which is a direct inhibitor of GPX4 [74].
  - Lovastatin: Lovastatin is another statin drug that can inhibit GPX4 and induce ferroptosis [78]. Similar to fluvastatin, the lovastatin target point is lipid synthesis, and it represents a HMGCR/HMG-CoA reductase inhibitor [79,80].
  - Gefitinib: As mentioned previously, it can be used to induce ferroptosis in TNBC [59].

After analyzing the genes that are affected by these drugs in this subgroup, we found that fluvastatin, lovastatin, and gefitinib downregulate CCT5, which was upregulated in this subgroup. CCT5 interacts with HMGCR, NFE2L2, and TP53. In the TP53 pathway, the upregulation of GLS2, which is a transcription target of TP53, leads to P53-dependent ferroptosis, and the inhibition of SC7A11 by TP53 can also trigger ferroptosis. TP53 (tumor protein 53) represses SLC7A11 to promote ferroptosis as a tumor suppression mechanism [81] (SLC7A11 was upregulated in this group). Fluvastatin and lovastatin bind to HMGCR and upregulate CDKN1A, which is a ferroptosis regulation gene [59].

c. *Subgroup3:* The patients in this subgroup are not Hispanic or Latino. They have right-sided TNBC as their neoplasm subdivision, they have less than three lymph nodes that are positive, and their histological type is an intraductal carcinoma. This subgroup contains 30 patients and has 118 unique DEGs that are abnormal in this subgroup, but not in the other subgroups (Tables S3 and S4). The drugs that can induce ferroptosis that are suggested for this subgroup are sunitinib and pazopanib.
  - Sunitinib: Sunitinib can induce ferroptosis by targeting the von Hippel-Lindau (VHL), which increases sensitivity to ferroptosis. Sunitinib interacted with 48 DEGs in this subgroup, including CDO1 [82]. The inhibition of CDO1 increases ROS and can induce ferroptosis [83].
  - Pazopanib: Pazopanib belongs to the same category as Sunitinib. Moreover, treating breast cancer cells with Pazopanib was found to induce autophagic cell death [84]. It is a necroptosis inhibitor [85]. The mTOR pathway is a regulator of iron metabolism. The VHL/HIF-α axis is the main regulator target of iron metabolism. HIF-3α was downregulated in this subgroup. Targeting the VHL gene pathway using drugs like sunitinib, sorafenib, pazopanib, and axitinib causes the VHL to be inactive. The inactivation of the VHL increases sensitivity to ferroptosis. IRP1 can bind to the iron reaction element of HIF-2α mRNA and inhibit its translation. Tempol, an IRP1-activated drug, inhibits HIF-2α and HIF-1α protein levels. PT2399 and PT2385 are inhibitors of HIF-2α [86]. Pazopanib is a tyrosine kinase inhibitor (TKI) that belongs to a class of drugs that targets PDGFR α/β and VEGFR activity [66]. PDGFR was found to be downregulated in this subgroup. After analyzing the genes in this subgroup, we found that sunitinib and pazopanib bind to FGFR2, which, in turn, interacts with HSPB1 and STAT3. FGFR2 was upregulated in this subgroup. Both of these genes play a role in ferroptosis induction, as explained previously. Moreover, sunitinib binds to PHKG2, which is important for the induction of ferroptosis [87,88,89].
d. *Subgroup4:* The patients in this subgroup are not Hispanic or Latino; they have left side TNBC as their Neoplasm Subdivision. They have less than three lymph nodes that are positive, and their histological type is an intraductal carcinoma. This subgroup has 32 patients and has 103 unique DEGs that are abnormal in this subgroup, but not in the other subgroups (Tables S3 and S4). The drugs that have the potential to induce ferroptosis in this subgroup are dexamethasone and vorinostat.
  - Dexamethasone: High-dose dexamethasone disrupts the metabolism of glutamate and cysteine, produces more ROS, and downregulates GPX4 and system XC−, which are two key mediators of ferroptosis [90]. In this subgroup, ROS1 was upregulated, GPX2–3 was downregulated, GPX5 was upregulated, and GPX4 was in the normal range.
  - Vorinostat: Vorinostat is a histone deacetylase (HDAC) inhibitor. In colon cancer cells, vorinostat can significantly inhibit cell growth and can induce cell cycle arrest and apoptosis [91]. It may induce ferroptosis by glutamine deprivation, resulting in the accumulation of ROS [92]. In NSCLC, vorinostat, combined with erastin, can increase the lipid peroxide levels and can inhibit HDAC to induce ferroptosis [93]. Moreover, when vorinostat is used as a combined therapy with gefitinib or erlotinib, which are EGFR-TKIs, it can promote oxidative stress-dependent apoptosis by the suppression of the c-MYC-regulated NRF2 functions and increase the levels of KEAP1 in NSCLC cells [94].

The analysis of this subgroup’s genes and drugs showed that dexamethasone bound to PTGS2, downregulated NFE2L2, and upregulated HSPB1, STAT3, and CDKN1A. These kinds of regulations promote ferroptosis. Dexamethasone downregulates GDF15 and NFKB2, and it upregulates MEGF9. In this subgroup, GDF15 was upregulated, NFKB2 was upregulated, and MEGF9 was downregulated. GDF15 interacts with ferroptosis-related genes like ATG7, EGFR, FTH1, HSPB1, PHKG2, PTGS2, AKR1C2, CDKN1A, DPP4, NFE2L2, and TP53. In this subgroup, AKR1C2 and DPP4 were downregulated. NFKB2, which was upregulated in this subgroup, interacted with EGFR, CDKN1A, and HSPA5. MEGF9, which was downregulated in this subgroup, interacted with GCLM, HMOX1, PTGS2, AKR1C2, which were downregulated in this subgroup; FDFT1 and NFE2L, which were upregulated in this subgroup; and GCLM. The downregulation of FTH1 can induce ferroptosis. IRP1 and IRP2, which were downregulated in this subgroup, are iron regulatory proteins that can regulate iron metabolism genes such as TFRC and FTH1 to maintain the stability of LIP [81]. In this subgroup, TFRC was upregulated while LIPC and LIPE were downregulated. AKR1C2, which was downregulated in this subgroup, is one of the FGRs. AKR1C1-3 (aldo-keto reductase family 1 member C1), which wase downregulated in this subgroup, is involved in eliminating end products of lipid peroxidation [59]. Vorinostat upregulates HMGCR, HMOX1, PHKG2, CDKN1A, and FDFT1.

e. *Subgroup5:* The patients in this subgroup are less than 50 years old; their pathological stage is m0 with no history of neoadjuvant treatment. This subgroup has 37 patients and has 216 unique DEGs that are abnormal in this subgroup, but not in the other subgroups (Tables S3 and S4). Opposite to what we observed in the other four subgroups, most of the top drugs that are suggested to this subgroup have an antioxidative effect on cancer cells. These drugs are rifampicin, galantamine, cerulenin, and lipoic acid.
  - Rifampicin: Rifampicin was repurposed as an antiferroptosis agent, and it functions as a lipid peroxyl radical scavenger [95,96].
  - Galantamine: Galantamine has an antioxidant effect and acethylcholinesterase and γ-secretase inhibitory activity [97].
  - Cerulenin: Cerulenin is an antifungal antibiotic that inhibits fatty acid and steroid biosynthesis. Fatty acid synthase (FAS) was observed to have a high expression in breast cancer cells in comparison with the normal cells, and there is increasing evidence that FAS plays a role in breast cancer development [98]. Moreover, fatty acid synthase (FAS) and ErbB2 have been shown to promote breast cancer cell migration [99]. The effect of cerulenin on breast cancer was tested in an in vivo analysis. Due to cerulenin’s ability to inhibit fatty acid synthase (FAS) that, in turn, decreases the expression of ErbB1, 2, and 4, it may initiate an epithelial-to-mesenchymal transition (EMT) as well as the migration and invasive ability of cancer cells [100].
  - Lipoic acid (LA): Lipoic acid is an antioxidant. It has an anti-proliferative effect in breast cancer cells by reducing breast cancer cell viability, cell cycle progression, and the EMT. It downregulates furin, which, in turn, inhibits the maturation of IGF-1R [101,102]. Combining lipoic acid with radiation therapy was found to overcome the resistance to radiation therapy and promote apoptosis [103].

### 3.2. Pathway Enrichment Analysis

**Figure 2:**
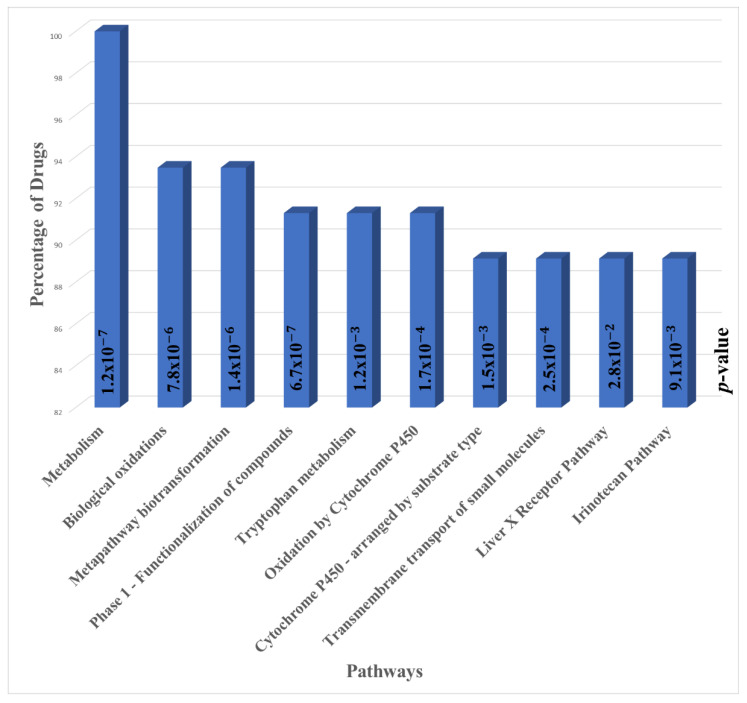
Pathways enrichment analysis for the genes targeted by top ten drugs for all the five subgroups of interest. The x-axis shows pathway names, and the y-axis shows the percentage of drugs that target each of these pathways.

We performed a pathways enrichment analysis in order to determine common mechanisms underlying the effects of the top ten drugs for each of the five subgroups (Table S5). After removing the duplication of drugs between subgroups, we identified 46 unique drugs in total for the five subgroups. We performed an enrichment analysis for pathways targeted by these drugs (Figure 2). The statistical significance of these pathways was calculated using Enrichr [104,105,106] with *p* < 0.05. The metabolism pathway was targeted by 100% of the drugs. Metabolic reprogramming is considered a hallmark of cancer that can be used for diagnosis, prognosis, and treatment [107]. Cancer cells show different metabolism patterns as compared with normal cells, and targeting that difference is a promising strategy for developing anticancer therapy [108]. Studies have shown that targeting metabolic enzymes can reverse drug resistance in cancer [109,110]. Targeting metabolism pathways could be an important component in developing a comprehensive treatment for breast cancer, considering the complexity of these pathways due to their crosstalk with other signaling pathways [111]. The upregulation of some metabolic pathways was found to stimulate metastasis in breast cancer [112]. The importance of targeting metabolism pathways also has been shown clearly in TNBC, where there is a metabolic heterogeneity between patients of this cancer subtype. The metabolism pathways were used to stratify TNBC patients into subgroups based on the heterogeneity of the metabolism [113,114]. Our findings coincide with what has been suggested in the literature and points to the importance of developing drugs that target metabolism pathways to tailor therapies for TNBC patients. The metabolic heterogeneity of TNBC was targeted by our top drugs, those directed at specific gene patterns contained within these subgroups. These patterns belong to the metabolism, but they were unique to a given subgroup and had high contrast with the outer population.

The second pathway that was targeted by 93% of the top drugs was the biological oxidation pathway. Increased oxidative stress plays a role in carcinogenesis in breast cancer patients [115]. In a study of the immune microenvironment of breast cancer, oxidative stress in combination with other biological phenotypes was associated with high immune infiltration [116]. Thus, targeting oxidation pathways could be promising for developing immune therapies for breast cancer. Studies have shown that the spread of BC metastatic cells and their survival in circulation depends on different factors, including antioxidant protection [117]. The oxidative stress response is used by breast cancer cells to adapt to their nutrient-poor environment [118]. Oxidative activity is one factor involved in the induction of breast carcinogenesis [119].

The metabolism and oxidation-related pathways can be observed more frequently than others among the pathways targeted by our top drugs. In analyzing the drugs, we focused on drugs that shared a common mechanism within the top five drugs. Still, the pathway enrichment analysis showed that the top ten drugs of all subgroups targeting metabolism and oxidation-related processes inhibit cancer growth and development. Moreover, we did an enrichment analysis for GO domains, cellular components, biological processes, and molecular functions. The top molecular functions that were highly enriched for the top ten drugs were oxidoreductase activity, protein homodimerization activity, and lipid binding. (Supplementary Figure S1 and Table S6). The top biological process enriched for the top drugs were the response to the drug, the oxidation-reduction process, and the response to the organonitrogen compound (Supplementary Figure S2 and Table S6). The top cellular components were the endoplasmic reticulum membrane, nuclear outer membrane–endoplasmic reticulum membrane network, and plasma membrane region (Supplementary Figure S3 and Table S6). 

### 3.3. Pharmacological Classes Analysis

We did a drug class enrichment analysis for the top ten drugs of the five subgroups of interest to find the pharmacological classes to which most of the drugs belong. Using the FDA Established Pharmacologic Class (EPC), we found classes for 32 drugs. The top pharmacological class to which most of these drugs belonged was the kinase inhibitor. Kinase inhibitors have shown great potential as a TNBC regimen due to their activity as antitumor and antiangiogenic therapies by targeting genes and pathways that play a role in cancer development and proliferation, like targeting EGFR, RON, MET, mTOR, BRAF, MEK, Src, and Bcr/Abl [120,121,122,123,124,125]. Kinase inhibitor drugs have been used in different breast cancer research and clinical trials, especially for TNBC, and they are considered one of the most successful targeted therapies for cancer [126,127]. They were used as a monotherapy and combined therapy for TNBC, and they are considered a promising strategy to treat advanced TNBC [128]. As a monotherapy, a tyrosine kinase inhibitor effectively inhibits the proliferation and induced autophagy and apoptosis in TNBC cells [129]. A kinase inhibitor was found to have the ability to preferentially suppress the growth and proliferation of TNBC cells in comparison with non-TNBC cells [130]. It was found to reduce the proliferation of mesenchymal stem-like (MSL) cells and induce apoptosis in TNBC cells as a combined therapy [131,132]. Moreover, a combination of kinase inhibitors was suggested for TNBC, where monotherapy has limited success [133]. Kinase inhibitors were used in combination with other inhibitors to overcome the resistance for monotherapy, like using dasatinib in combination with Akt or mTOR inhibitors to overcome dasatinib resistance and reduce the proliferation in TNBC cells [134]. Many kinase inhibitors were effective as a TNBC therapy, and many others are a subject of ongoing clinical trials [135,136].

## 4. Discussion

Breast cancer is a highly heterogeneous disease. Its heterogeneity requires regimens to be tailored for homogeneous subgroups of patients with common phenotypic and genotypic features that are unique to each subgroup. This study applied our data-driven approach to stratify breast cancer into subgroups using their phenotypic and genotypic data. The method starts with the phenotypic features and partitions the disease population based on the categorical values of the phenotypic variables. This partitioning created different subgroups. For each subgroup, the algorithm used the genotypic features to evaluate the druggability potential, which refers to the necessity of treating a given subgroup differently and tailoring drugs to that subgroup. 

Since developing a de novo drug is expensive and time-consuming, we developed a computational method to reposition drugs for each subgroup based on the gene expression patterns and the relationship between these genes and other biomedical entities like pathways and GO domains. Each subgroup’s genotypic features are represented as a network and processed using a graph database, neo4j. Each network consists of heterogeneous biomedical entities. The core of each network is the differentially expressed gene patterns in that subgroup. For each gene, the network contains other genes that directly interact with that gene, the pathways and GO domains to which that gene belongs, and the drugs that affect the expression of the other genes in that network. The opposite expression regulation between the drugs and the affected genes is taken into account by retrieving the drugs from the knowledgebase into the subgroup’s network using cMAP data. Each network has several drugs, and to find the best drugs that can be suggested for a given subgroup, we developed a method to rank the drugs within each network using different factors. These factors include the percentage of differentially expressed genes, the number of patterns targeted by each drug, and the perturbation impact of a drug on human cells. 

Breast cancer has three major subtypes, luminal A, luminal B, and triple-negative breast cancer (TNBC). TNBC patients have the lowest survival rates among all breast cancer patients. This motivated us to focus our study on TNBC patients. We analyzed five subgroups composed of combinations of the three population variables, which are age, race, and neoplasm subdivision, as statistically significant phenotypic variables. After analyzing the differentially expressed genes for these subgroups, we found these subgroups had, on average, 162 unique differentially expressed genes in addition to phenotypic-level differences. This uniqueness does not only mean the gene is differentially expressed in a given subgroup, but it also could mean the gene has a differential expression pattern unique to a given subgroup. It could be only upregulated in that subgroup while it is downregulated in the other subgroups, and vice versa. 

Analyzing the top five drugs for the subgroups of interest showed a common mechanism shared by four subgroups, with race and neoplasm subdivision as the significant phenotypic factors. Still, we found the opposite mechanism was suggested for the subgroup with age as a significant phenotypic factor. Inducing ferroptosis was the common mechanism in the subgroups that were Black or African American, were White, had right side breast cancer, or the subgroups with left side breast cancer. In contrast, the antioxidant was the common targeted mechanism in the subgroup with patients aged less than 50 years. This finding coincides with what has been found previously [59], where age is negatively correlated with ferroptosis. The pathway enrichment analysis also showed that the most targeted pathways by the top ten drugs were pathways that have a relation to metabolism and oxidation. The kinase inhibitor is considered one of the most targeted therapies to treat breast cancer, especially TNBC. Since it is a computational-based method, a literature review was used to validate our findings. Still, these findings need more testing in a wet lab setting to ensure it is valid to be suggested for healthcare improvement. After validation in the wet lab, these results can significantly improve TNBC survival and suggests drugs tailored for a specific group of patients based on their phenotypic and genotypic features. 

**Figure 3:**
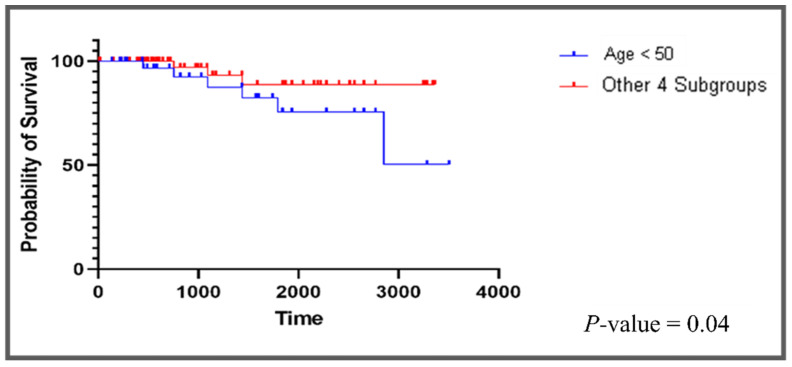
Survival curves for subgroup 5 vs. other subgroups. The curve shows the patients younger than 50 who have worse survival rates than the other 4 subgroups of interest have.

The clinical relevance of our data regarding repurposing TKIs as ferroptosis inducers is strengthened by recent findings based on the functional analysis of breast cancer cell lines that demonstrate that TNBCs are enriched in ferroptosis gene signatures and are vulnerable to ferroptosis inducers, compared to other breast cancer subtypes, i.e., ER-positive and/or HER2-positive subtypes [137]. Furthermore, age is the most pertinent clinical characteristic that has been shown to have a negative correlation with ferroptosis in certain tumors like BC, i.e., in younger BC patients, ferroptosis is less clinically relevant [59] which coincides with our discovery of a completely different class of repurposed medications that did not induce ferroptosis in our younger (<50 years) cohort of BC patients. The potential benefit from antioxidants in younger TNBC patients, as we have demonstrated in our current bioinformatic analysis for patients younger than 50 years, is further supported by biological data on the dependence on reactive oxygen species (ROS) for the survival and malignant progression of TNBC in younger patients. These high levels of ROS production that drive the aggressiveness in TNBC result from oncogenic gene mutations, e.g., BRCA1 [138], gene expression changes, e.g., BLT2 [139], and the attainment of stem-cell like properties [140]. This high ROS content induces multiple signaling which, in turn, leads to highly proliferative, migratory, and drug-resistant phenotypes in TNBC and is effectively attenuated by antioxidants, thereby reducing the growth and metastasis of TNBC cells [141]. The shorter survival observed in the younger cohort or subgroup 5 when it was compared against all remaining four subgroups “bundled” together (Figure 3) is a testament to the different ROS-dependent biology in subgroup 5, which contributes to the cell survival, metastasis, chemoresistance, and cancer relapse. Therefore, the additional use of antioxidants can be an effective strategy for treating these younger TNBC breast cancer patients in order to prevent chemoresistance through depleting ROS.

The limitation of this data-driven research is threefold: firstly, the selection of thresholds for support and growth is dependent on the availability of data and the research question to be answered. Variations will affect the resulting number of patterns and the subgroups. Secondly, while this paper is focused on TNBC using a foundation developed for colorectal cancer [37], a pan-cancer study is needed to further test the robustness of the methods in all major cancers. Thirdly, further studies are required in a “wet lab” in order to validate our data and monitor the efficacy of repurposed drugs suggested, like TKIs and antioxidants. For the use of these proposed medications to be clinically relevant, further studies will need to examine their potential interference with standard chemotherapy, the timing of administration relative to standard chemotherapy, and the appropriate treatment duration. 

## 5. Conclusions

Breast cancer is a heterogeneous disease. Tailoring drugs for homogeneous subgroups of patients is necessary to improve survival, but developing de novo drugs is an expensive, time-consuming, and high-risk process. Drug repositioning represents a promising direction to address this problem by taking advantage of already FDA-approved drugs and using them to treat additional diseases. We implemented our subgroup stratification and drug repositioning method on breast cancer data. Our analysis focused on TNBC subgroups because this subtype has the worst survival rate among breast cancer patients. For this study, we analyzed five TNBC subgroups. These subgroups have age, race, and neoplasm subtype as significant phenotypic variables and had differentially expressed genes unique to each subgroup. The analysis of these subgroups and the suggested drugs showed that developing drugs that target antioxidants and ferroptosis could be a promising direction to tailor drugs for TNBC patients. The analysis of the top drugs suggested for each subgroup showed that either these drugs are used to treat cancer or are subject to ongoing research to be used for cancer. Most of the recommended top ten drugs for all the subgroups belongs to the kinase inhibitors class. This class of drugs is one of the well-established target therapies for treating cancer. Many inhibitors are subject to clinical trials as a regimen for breast cancer or TNBC. Validating our findings in a wet lab will be a step toward improving TNBC healthcare without developing de novo drugs, which will save costs, time, and human lives.

## Data Availability

The data is open source, and the results are listed in the supplement document.

## Supplementary Materials

The following are available online at https://www.mdpi.com/article/10.3390/cancers13246278/s1, Table S1: Phenotypic variable categories, Table S2: The relationships between the biomedical entities in drug repositioning knowledge base (BR-KB) and the source databases, Table S3: The subgroups of interest and the differentially expressed genes in each subgroup exclusively, Table S4: The statistical properties of the five subgroups of interest, Table S5: Top ten drugs for each of the five TNBC subgroups of interest, Table S6: Biomedical entities enrichment analysis, Figure S1: Top molecular functions targeted by top ten drug of the five subgroups of interest, Figure S2: Top biological processes targeted by top ten drug of the five subgroups of interest, Figure S3: Top cellular components targeted by top ten drug of the five subgroups of interest.
