# Supplementary material for: Drug Repositioning and Subgroup Discovery for Precision Medicine Implementation in Triple Negative Breast Cancer
Source: Cancers (Basel). 2021 Dec 14;13(24):6278. doi: 10.3390/cancers13246278 (PMC8699385; doi:10.3390/cancers13246278)
Supplement: Supplementary file 1 [file cancers-13-06278-s001.zip › cancers-1397615-supplementary.pdf]

Supplementary Materials

Drug Repositioning and Subgroup Discovery for Precision Medicine Implementation in Triple Negative Breast Cancer

Zainab Al-Taie, Mark Hannink, Jonathan Mitchem, Christos Papageorgiou and Chi-Ren Shyu

Table S1: Phenotypic variable categories

	Variable	Categories	Count
**1**	**Age (26-90)**	<50	265
		>=60	459
		50s	256
**2**	**ER Level Cell Percentage Category**	0-49%	104
		50-99%	302
		None	574
**3**	**Histological Type**	IDC	702
		ILC	182
		infiltrating carcinoma nos	1
		MED	6
		metaplastic carcinoma	10
		MIXED	25
		MUC	15
		OTHER	39
**4**	**History of Neoadjuvant Treatment**	NA	1
		no	972
		yes	7
**5**	**Lymph Node Examined Count**	<=3	252
		>=10	429
		4-9	195
		None	104
**6**	**Margin Status**	close	28
		NA	64
		negative	888
**7**	**Menopause Status**	indeterminate	26
		NA	72
		peri	36
		post	642
		pre	204
**8**	**Neoplasm Subdivision**	left	508
		right	472
**9**	**Number of Lymph Nodes Positive**	<=3	691
		>=10	54
		4--9	91
		None	144
**10**	**Patient Ethnicity**	hispanic or latino	34
		NA	155
		not hispanic or latino	791
**11**	**Patient Race**	american indian or alaska native	1
		asian	59

		black or african american	156
		NA	81
		white	683
**12**	**Percent Lymphocyte Infiltration**	>40%	16
		0-9%	297
		10-39%	71
		None	596
**13**	**PR Level Cell Percent Category**	0-49%	193
		50-99%	182
		None	605
**14**	**Stage**	advanced	152
		nx	6
		primary	822
**15**	**Stage m**	m0	828
		mx	152
**16**	**Stage n**	n0	483
		n1	323
		n2	100
		n3	62
		nx	12
**17**	**Stage t**	t1	259
		t2	572
		t3	118
		t4	28
		tx	3
**18**	**Subtype**	HER2+	32
		Luminal_A	392
		Luminal_B	113
		NA	47
		Others	287
		TN	109
**19**	**Tumor Necrosis Percent**	0	556
		1-10	215
		11-20	103
		21-30	104
		None	2

Table S2. The relationships between the biomedical entities in drug repositioning knowledge base (BR-KB) and the source databases from which these entities were curated.

**Metaedge**	**Source**
Anatomy–downregulates–Gene	Bgee
Anatomy–expresses–Gene	TISSUES, Bgee
Anatomy–upregulates–Gene	Bgee
Compound–binds–Gene	DrugCentral, BindingDB, DrugBank
Compound–causes–SideEffect	SIDER
Compound–downregulates–Gene	LINCS L1000
Compound–palliates–Disease	LabeledIn, MEDI, PREDICT, ehrlink
Compound–resembles–Compound	DrugBank
Compound–treats–Disease	LabeledIn, MEDI, PREDICT, ehrlink
Compound–upregulates–Gene	LINCS L1000

Disease–associates–Gene	DISEASES, GWAS Catalog, DisGeNET, DOAF, hetio-dag
Disease–downregulates–Gene	STARGEO
Disease–localizes–Anatomy	MEDLINE
Disease–presents–Symptom	MEDLINE
Disease–resembles–Disease	MEDLINE
Disease–upregulates–Gene	STARGEO
Gene–covaries–Gene	Evolutionary Rate Covariation
Gene–interacts–Gene	hetio-dag, Incomplete Interactome, Human Interactome Database
Gene–participates–Biological Process	Gene Ontology
Gene–participates–Cellular Component	Gene Ontology
Gene–participates–Molecular Function	Gene Ontology
Gene–participates–Pathway	Pathway Interaction Database, Reactome, WikiPathways
Gene-regulates-Gene	LINCS L1000
PharmacologicClass–includes–Compound	DrugCentral[3]

(**Note**: all the preprocessing for the source data can be found in [45]).

Table S3: The subgroups of interest and the differentially expressed genes in each subgroup exclusively.

Subgroup ID	Subgroup Description	Uniquely Differentially Expressed Genes
Subgroup1	Black or African American with no history of neoadjuvant treatment	MKL2 , KIAA0232 , ZNF132 , ARHGAP26 , PHF17 , SERINC1 , TMEM144 , MTUS1 , VPS13C , ZNF175 , C14orf45 , ZNF763 , FGD4 , PEX11A , NUDT16P1 , ABLIM1 , SBF2 , PCDHGA11 , ARSG , RALGAPA2 , SPATA6 , FNIP2 , SALL2 , BNIP3L , SAMD8 , SPEF2 , MYO9A , EGFR , DCUN1D3 , CALN1 , ZNF660 , ANXA1 , KCNAB1 , LMBRD2 , FOXI2 , CNTN4 , GATA2 , MPP2 , NAALADL2 , RGPD5 , LRRC37A2 , LOC440925 , RNF150 , HADH , PDP2 , OLFM1 , GRIN2B , LOC162632 , FBXO15 , EPHA6 , TMOD1 , PRINS , LNPEP , SERPINA5 , SLC16A6 , ST8SIA6 , STOX1 , GUCY1A2 , RHBDL3 , GATA3 , C1orf175 , NOS1AP , HOXD10 , LOC342346 , SV2B , C11orf53 , BAGE2 , ALS2CR12 , EYA1 , SP5 , ANKRD31 , FAIM2 , SLC4A8 , CCDC148 , ADAMTSL3 , LOC400804 , NPFFR2 , RASEF , POF1B , CSMD1 , CRTAC1 , RBM20 , ME1 , CNGA3 , MOGAT1 , GABRE , UPF0639 , NR0B1 , MS4A8B , C8orf86 , ADARB2 , ADCY10 , FAM184B , C9orf4 , ASCL1 , GPR128 , PSG4 , BOLA2 , PWP2 , MGAT4B , MTERFD1 , PGAM5 , SCO2 , ZC3H3 , SOLH , GRK6 , UNC93B1 , FKBPL , ZNF707 , MRPL4 , MRPL15 , THOC6 , NFKBIB , DTX2 , RGS19 , MTFR1 , DUSP23 , PTMS , CPSF1 , TIMM44 , RPS6KA4 , ASPSCR1 , STK32C , GUK1 , C17orf90 , ANKRD13B , HAUS7 , PLEKHO1 , EXOSC5 , SSR4 , CTU2 , DCBLD1 , LOC541471 , OCIAD2 , TSPO , NTHL1 , NRAS , PARP10 , SNHG6 , GLI4 , S100A16 , FJX1 , PPP1R16A , CCDC124 , RGS14 , WNT7A , KCNK10 , UFSP1 , SPSB2 , FAM128B , PHTF1 , KLF16 , RPSAP52 , FKBP11 , RAC2 , DNLZ , TLR9 , DEF6 , LAT , KIAA0802 , 1-Sep , CD79B , SLC16A1 , RASAL3 , HSP90B3P , RAB33A , PBX4 , RUNX3 , CTCFL , TRIM69 , WAS , NACA2 , OR7E156P , KHDC1L , CIITA , SP8 , SHISA3 , ADAMTSL2 , TMC8 , TOX , CACNA1A , POU3F4 , SOX15 , PARVG , FERMT1 , TMEM121 , C19orf38 , UNC5CL , HLA-DRB1 , FERMT3 , SPI1 , SCRT2 , BSND , RASL11B , TGFBI , HCK , MC5R , MYO7B , CARD9 , C20orf134 , CCDC88B , SHD , EBI3 , COL27A1 , LOC100130331 , ITGB2 , MEF2B , NAPSB , CHMP4C , CPNE5 , CRYBB2 , LOC100132724 , TTBK1 , LPPR5 , LAX1 , TRPV4 , LILRB2 , MAL , POU3F1 , TSPAN10 , PDE6G , C20orf103 , RENBP , GPR85 , SULT1E1 , REG4 , GJD4 , FOSL1 , FXYD7 , SLAMF1 , SIGLEC7 , VSIG8 , CDH4 , BNC1 , POM121L2 , SYCE1L , ANP32C , CCDC154 , SLC6A12 , FLJ40330 , APOE , KRT7 , CCL22 , TNFSF9 , DUSP5P , ZNF679 , IL1A , RPL31P11 , TUBBP5 , GPR64 , KLK1 , MYH15 , KIAA0125 , ERAS , NELL1 , TUBB4Q , PHOX2A , MIXL1 , KLKP1 , KRT19 , FGFBP1 , CEBPE , GBP6 , PRMT8 , KLK4 , RHOV , EMR3 , HSD17B3 , DSG3 , COL6A4P2 , BEND4 , C19orf21 , PROK2 , HES5 , ERN2 , HBE1 , TBX4 , NEFM , LILRA4 , MUC7 , NXPH2 , KLK11 , DDC , JSRP1
Subgroup2	Hispanic or Latino and White; they have less than three positive lymph nodes, and their histological type is intraductal carcinoma	FLJ10357 , NEAT1 , LTC4S , STARD8 , PRDM8 , ZNF671 , CELF6 , ADCY6 , DNAH1 , MYO15B , ARHGEF37 , SEC31B , KIAA1462 , LOC390595 , PHLDB1 , LOC648740 , GPR17 , HIPK4 , SSC5D , ZDBF2 , C19orf36 , SLC16A11 , LOC283731 , ADAMTSL4 , RASGRP4 , MST1P9 , CYP26B1 , C15orf59 , WNT11 , PRSS45 , PRELP , KRT1 , RIC3 , FLJ46111 , ZMYND17 , CARNS1 , RNASE13 , LAMC3 , LOC554202 , KRT14 , OBSCN , TM4SF19 , SYCE1 , PITX3 , CCT5 , INTS7 , PDIA6 , FBXO45 , P4HB , CENPH , PRPS2 , FBXO22OS , SKP2 , HYLS1 , FLVCR1 , MBOAT2 , C17orf96 , AADAT , ZNF713 , EFR3B , FAM27C , EGLN3 , MAGEA5 , SPINT2 , CALCA , SMOC1 , LOC100133985 , FLJ30679 , C14orf72 , TRPC4 , FAM27A , PRSS8 , XKR9 , FAM71F1 , FAM83B , C9orf135 , SLC16A10 , C21orf62 , SLC34A2 , C11orf85 , SPSB4 , UGT2B7 , HGC6.3 , NR1H4 , PGPEP1L
Subgroup3	Hispanic or Latino, right side TNBC as their Neoplasm Subdivision, less than 3	SLC7A8 , MAP6 , C1orf21 , ZC3H12B , ATP1B2 , BTD , ZCWPW2 , NACAD , ZBTB7C , LOC644538 , LOC285593 , RYR3 , GNA14 , LONRF3 , CYP11A1 , C2orf58 , OTUD7A , C14orf64 , KIF17 , ADRA2C , THPO , PURG , RGN , ACOT1 , HTR7 , ART5 , ROPN1L , REEP6 , ANKRD20A3 , FSTL3 , C6orf186 , OTUD1 , SCNN1A , ADAM23 , PTGFR , SEC14L2 , CH25H , TCF15 , FASN , SLC44A4 , LDHAL6A , PRKAA2 , SFTPA2 , CHST8 , C10orf107 , LOC440173 , KLF14 , LOC100302650 , C4BPB , KCNS2 , LOC285205 , FFAR2 ,

	lymph nodes positive, and the histological type is intraductal carcinoma	PPBP , SPINK5 , EREG , AMHR2 , PHKG1 , NAT2 , ZP1 , ALDH3B2 , TRIM55 , FCGR3B , CEACAM5 , MYO19 , GEMIN8P4 , SULF1 , PRTFDC1 , TRPM5 , CBX8 , CDK18 , MATN1 , SNORD1C , IL1F7 , OAS3 , SPIC , LGALS9 , ARHGEF16 , CYP2S1 , RAGE , TNFSF4 , C9orf172 , LRRC69 , GFI1B , ADAM21 , FAM176A , CCR5 , GPR141 , PIRT , CD274 , GRIK2 , LOC84989 , ITK , ELFN2 , TMEM200C , VSNL1 , CTNNA2 , GPRIN2 , C12orf59 , SH2D4B , MEGF11 , QRFP , NOG , SIGLEC14 , FGFR2 , IKZF3 , CDHR5 , GNAT2 , BTLA , FXYD3 , BCMO1 , ZNF80 , FPR2 , LDHC , DKK4 , T , DKFZp434J0226 , DAPL1 , TIMD4
Subgroup4	Not Hispanic or Latino, left side TNBC, less than three lymph nodes positive, and the histological type is intraductal carcinoma	RBM43 , TRIM66 , ALS2CR8 , GLT8D2 , STARD13 , MEGF9 , HERC2P2 , SUGT1L1 , ANKAR , RGL1 , ZNF844 , CYYR1 , STX1B , ETV1 , PDGFRA , FLJ42709 , ZNF662 , RGAG1 , KAL1 , C1orf183 , KLF10 , FER , PER3 , HMCN1 , RCAN2 , PCDHB19P , FLRT3 , DLEC1 , GPR62 , LPAR4 , MECOM , MCTP1 , MT1M , KIF5C , REEP1 , PIP5K1B , EFNB3 , SYT1 , DIRAS2 , ZBTB20 , KCNIP1 , FRZB , NFATC2 , LOC153328 , ADAM22 , CCDC144A , FRMPD4 , MN1 , SLC28A3 , TPH1 , GAMT , DBC1 , C12orf53 , PCDH20 , TSSK1B , KCNJ11 , KCNH3 , GOLGA8C , CLTCL1 , P2RY4 , MUC4 , SHISA2 , SLC27A6 , CHST9 , XKR4 , CCL13 , FAM55B , ISL1 , SMR3B , TMEM233 , DGKB , ASB16 , NXF3 , LOC647859 , LALBA , MAGOH , C16orf61 , TBRG4 , PTS , NFKB2 , HLA-B , MTSS1L , PSMB10 , MBLAC1 , TMED3 , LGALS9C , APOB48R , FAM178B , CLDN4 , CDH3 , GDF15 , WBSCR26 , LOC100129066 , C15orf48 , KIRREL3 , GGTLC2 , DOK7 , LOC440896 , SLC5A11 , DLK1
Subgroup5	Age is less than 50 years old; stage is m0, and history of neoadjuvant treatment	CYB5D2 , CCND2 , PTGER4 , PECI , PPP4R1L , C4orf38 , GIMAP7 , JAKMIP3 , PCDHB4 , INHBB , C12orf63 , AMDHD1 , PCDHA10 , TSHR , SCGN , ADAMTSL5 , OR52N4 , CRYM , TPSB2 , AADACL3 , LOC643719 , MAP3K15 , TDRD9 , LHFPL4 , TPSAB1 , TKTL1 , CEACAM7 , HS3ST2 , IFNE , CETN4P , PCP2 , KNDC1 , KIF25 , CELF5 , SCGBL , TARP , UPK3A , TFAP2B , ACHE , ARX , C17orf77 , GRP , ABCC12 , CELP , GLYATL3 , MRPL13 , CCDC99 , PSMG1 , MANF , OPN3 , RDH12 , SMO , GPX7 , ATP5J2 , SLC5A6 , SLC43A3 , LEPRE1 , C1orf122 , C19orf57 , ELOVL4 , PNPLA1 , C1orf105 , VNN1 , POLR3G , C5orf13 , CCDC18 , ARL14 , MAL2 , FKBP10 , PSIMCT-1 , TIMP1 , SOAT1 , MOCOS , HIST1H2AL , FAM19A5 , CRYBA2 , CRCT1 , SPRR2G , LOC100144603 , LOR , SPINK6 , PALM2 , SLC13A3 , PLA2G2F , C9orf70 , CNFN , LCE3D , GNLY , LOC100190940 , ACP5 , C4orf26 , DGAT2L6 , FAM84A , SNHG9 , ABCC13 , ADAMTS20 , HIST1H4I , PRG2 , GPR63 , ACOT11 , KRTDAP , SPRR4 , LCE3E , FAM81A , PON1 , MC4R , HBQ1 , AWAT1 , CD180 , LOC400940 , OLFML2B , FAM176B , RUNDC3A , SLC12A5 , ALOX12 , SEZ6L2 , KCNH6 , KPRP , SYT4 , LY6H , CALHM3 , MYT1 , PTP4A3 , C21orf56 , XKR3 , S1PR5 , SPRR2E , PTCHD2 , C20orf123 , NEUROD4 , NR1I2 , FAM123C , EMILIN1 , FAM25A , PEG3 , MORC1 , HHLA2 , VTCN1 , PCOLCE , C13orf16 , GPAT2 , CCL26 , CAMK1G , NUP210L , MED12L , LCE1C , SVOP , CCDC129 , SEZ6 , KCNE1L , KLK13 , TRIM67 , PTPRN , CASKIN1 , KIR2DL3 , FAM163A , PCSK2 , CREG2 , SPRR2B , CBLN2 , FA2H , PGA5 , TMEM155 , LSAMP , TDRD1 , ACSBG1 , BTN1A1 , EMX1 , EVPLL , NRSN1 , CKB , SAGE1 , ZNF536 , IGF2 , ZIM2 , KCNJ3 , CMTM5 , RIMS4 , ARID3C , GPR6 , BEX1 , SPRR2A , NBPF4 , ADAMTS19 , CDSN , C1orf125 , HMP19 , FAM3D , OLFM4 , ABHD12B , TMEM211 , LOC286002 , RFPL1 , KIR2DL1 , SST , PHF21B , HMX1 , CSN1S1 , C10orf99 , RFPL4B , SPRR1A , ANKRD26P1 , ACER1 , GRID2 , C3orf72 , CHRNA4 , LHX8 , CSMD3 , VSTM2A , CST5 , CGA

Table S4. The statistical properties of the five subgroups of interest.

Subgroup ID	Population Size	Support		Growth		Lift		Chisq		Confidence
		Min Support	Max Support	Min Growth	Max Growth	Min Lift	Max Lift	Min Chisq	Max Chisq	
**Subgroup1**	**30**	0.70	0.97	1.532258034	12.78846169	1.507692337	9.397260666	5.970108986	166.4014587	1
Subgroup2	37	0.70	0.95	1.781313539	9.148995399	1.730273008	6.996430397	12.77438164	160.7710266	1
Subgroup3	30	0.70	0.97	1.852836847	10.90163898	1.805695176	8.36585331	12.37418842	147.271225	1
Subgroup4	32	0.72	1	1.751606703	10.48269272	1.709648013	8.004261017	10.85119724	152.2507629	1
Subgroup5	37	0.70	0.95	1.825478315	12.28812695	1.770305037	8.616086006	13.71959972	197.8222656	1

Each row is for one of the subgroups. Each column is a data mining metric and statistical properties for that subgroup. The column "Subgroups ID" is for the IDs given to each of the subgroup in the paper. "Population Size" is the number of patients in each subgroup. "Support" is the minimum and maximum support for the genotypic patterns in that subgroup. "Growth" is the minimum and maximum growth rate for the genotypic patterns in that subgroup. "Lift" is the minimum and maximum lift of the genotypic patterns in that subgroup. "Chisq" is the minimum and maximum Chi-squared test for the genotypic patterns in that subgroup. The "Confidence" is the confidence factor for genotypic patterns of each subgroup.

Table S5. Top ten drugs for each of the five TNBC subgroups of interest.

**Top 10 Drugs for Five TNBC Subgroups of Interest**					
**ID**	**Subgroup1**	**Subgroup2**	**Subgroup3**	**Subgroup4**	**Subgroup5**
1	Digitoxin	Homoharringtonine	Sunitinib	Idarubicin	Rifampicin
2	Ouabain	Chenodeoxycholic acid	Pazopanib	Topotecan	Varenicline
3	Digoxin	Fluvastatin	Mycophenolic acid	Mitomycin	Tubocurarine
4	Daunorubicin	Lovastatin	Lurasidone	Vinblastine	Cerulenin
5	Afatinib	Gefitinib	Mianserin	Gemcitabine	Galantamine
6	Bosutinib	Bosutinib	Mirtazapine	Tolazamide	Sorafenib
7	Lapatinib	Erlotinib	Risperidone	Levetiracetam	Topotecan
8	Gefitinib	Cyclosporine	Asenapine	Epirubicin	Vemurafenib
9	Tretinoin	Sunitinib	Cabergoline	Irinotecan	Methylphenobarbital
10	Mebendazole	Mycophenolic acid	Iloperidone	Nilotinib	Primidone

Table S6: Biomedical Entities Enrichment Analysis

ID	Pathways		Molecular Function		Cellular component	
	**Object Name**	**%**	**Object Name**	**%**	**Object Name**	**%**
1	Metabolism	100.0	oxidoreductase activity	97.8	endoplasmic reticulum membrane	97.8
2	Biological oxidations	93.5	protein homodimerization activity	95.7	nuclear outer membrane-endoplasmic reticulum membrane network	97.8
3	Metapathway biotransformation	93.5	lipid binding	95.7	plasma membrane region	97.8
4	Phase 1 - Functionalization of compounds	91.3	iron ion binding	95.7	synapse	95.7
5	Metabolism of lipids and lipoproteins	91.3	oxidoreductase activity_ acting on paired donors_ with incorporation or reduction of molecular oxygen	95.7	receptor complex	91.3
6	Tryptophan metabolism	91.3	steroid binding	93.5	apical part of cell	91.3
7	Oxidation by Cytochrome P450	91.3	heme binding	93.5	neuron projection	91.3
8	Cytochrome P450 - arranged by substrate type	89.1	tetrapyrrole binding	93.5	vesicle membrane	89.1
9	Transmembrane transport of small molecules	89.1	transmembrane transporter activity	91.3	secretory vesicle	89.1
10	Constitutive Androstane Receptor Pathway	89.1	drug binding	91.3	cell body	89.1
11	Liver X Receptor Pathway	89.1	substrate-specific transmembrane transporter activity	89.1	axon	89.1
12	Irinotecan Pathway	89.1	monooxygenase activity	89.1	neuronal cell body	89.1

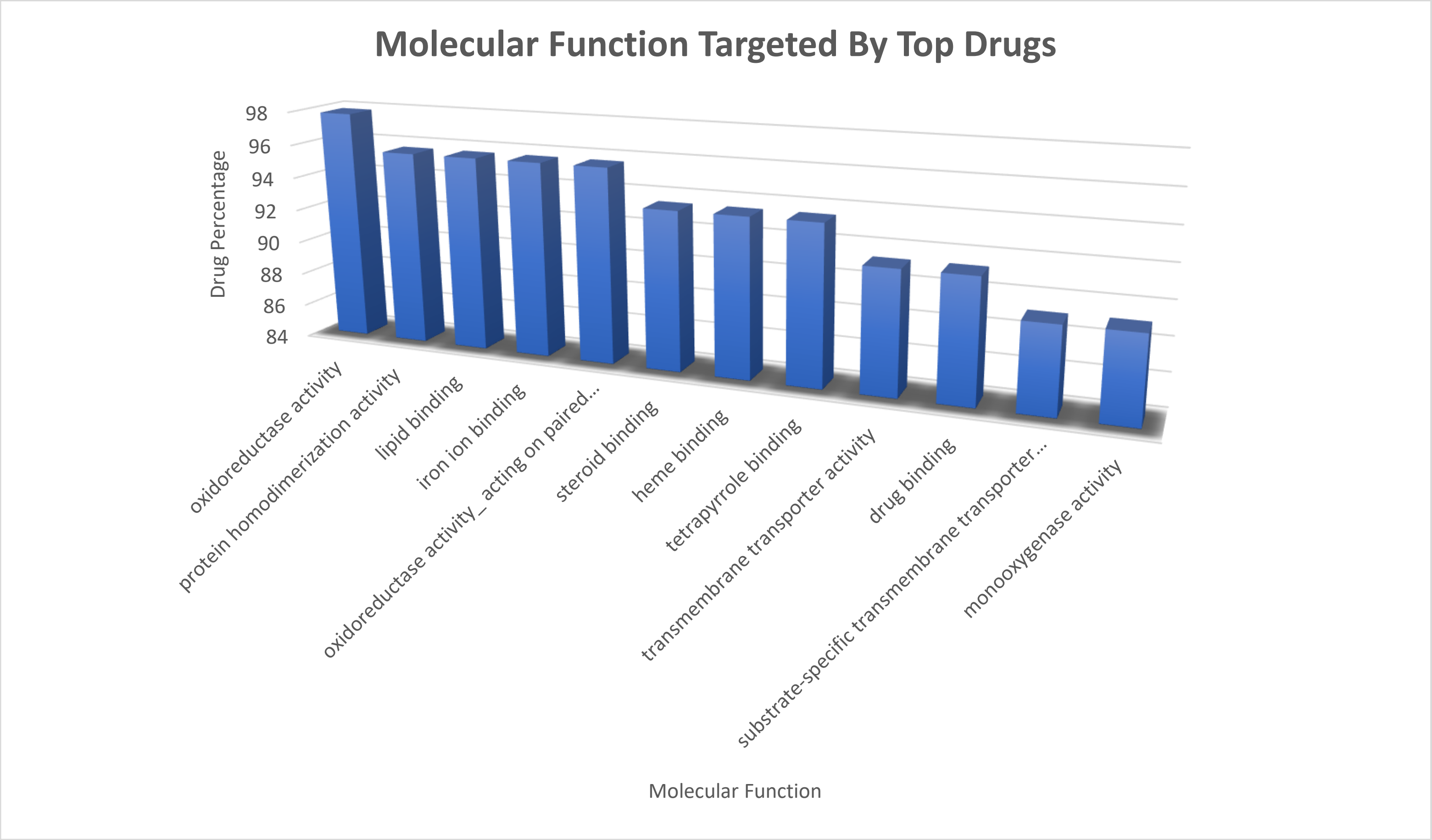

Figure S1: Top molecular functions targeted by top ten drug of the five subgroups of interest.

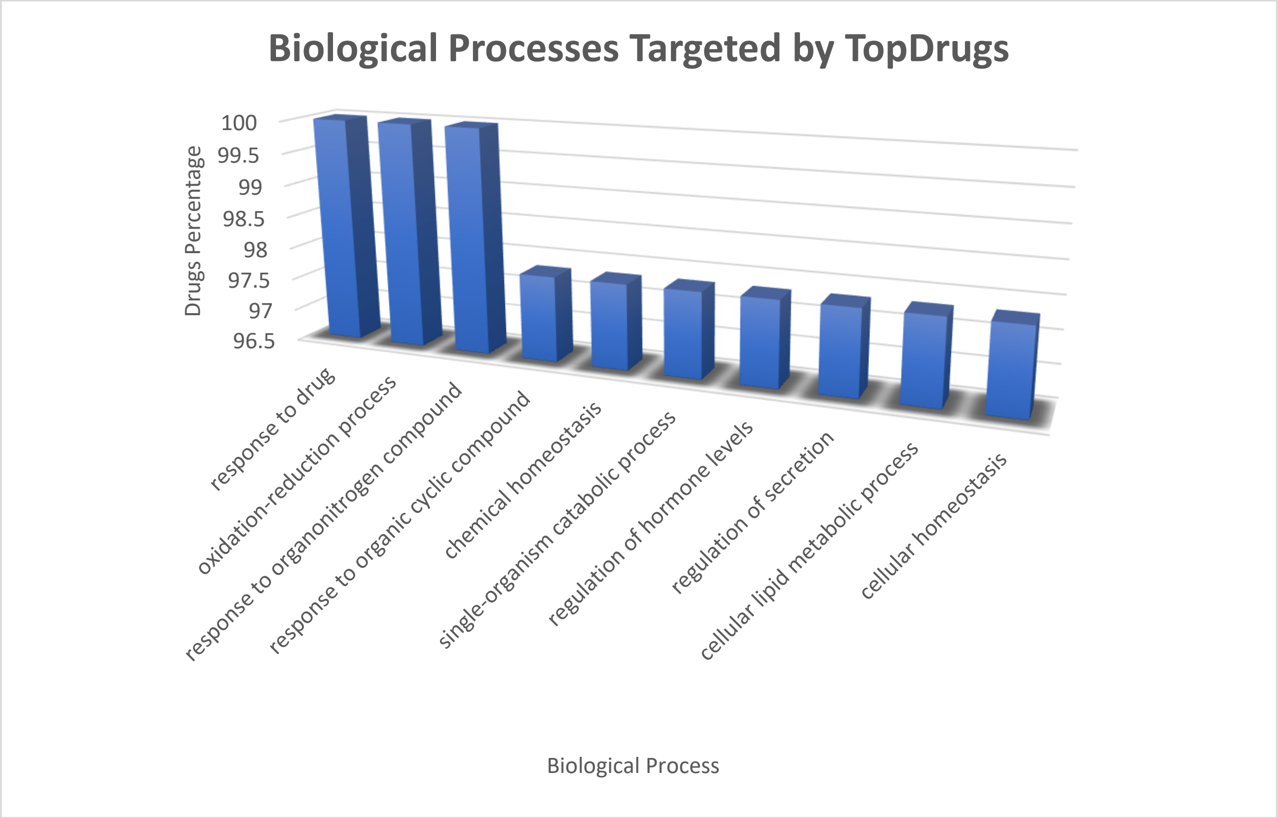

Figure S2: Top biological processes targeted by top ten drug of the five subgroups of interest.

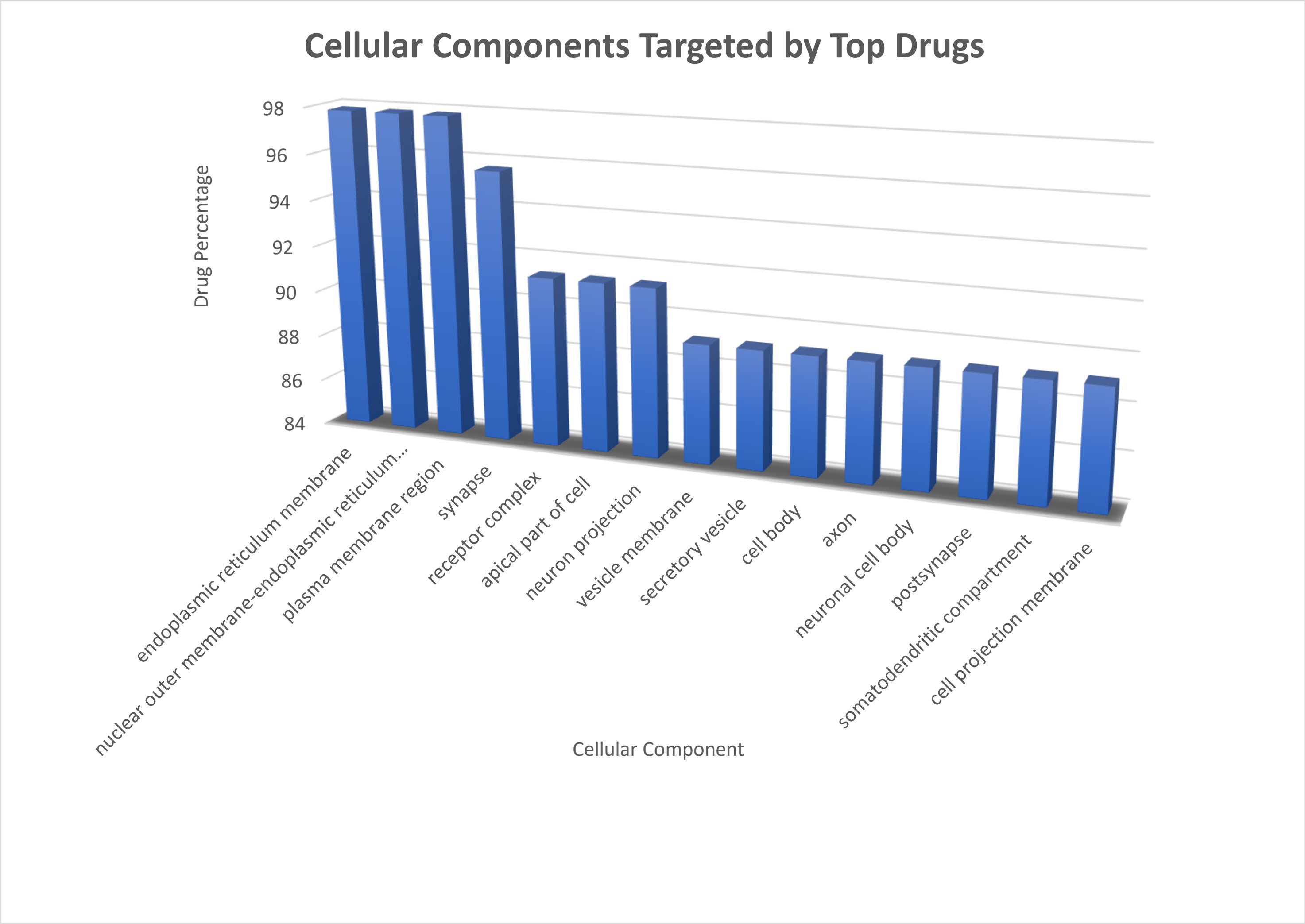

Figure S3: Top cellular components targeted by top ten drug of the five subgroups of interest.
